# Neuroendocrine carcinoma of the colon presenting as acute meningitis

**DOI:** 10.1186/s12883-019-1310-x

**Published:** 2019-05-01

**Authors:** Julie R. Bloom, Arlen Brickman, Fan J. Yang, Ji-Weon Park, Jonathan Cheponis

**Affiliations:** 1 Rush University Medical Center, Chicago, IL USA; 2 Department of Neurological Sciences, Rush University Medical Center, Chicago, IL USA

**Keywords:** Neuroendocrine tumor, Gastroenteropancreatic neuroendocrine tumor, Carcinomatous meningitis, Leptomeningeal disease, Metastatic disease, Cerebrospinal fluid

## Abstract

**Background:**

Neuroendocrine tumors represent an expansive group of neoplasms that share an etiology of epithelial origin with neuroendocrine differentiation. Poorly-differentiated neuroendocrine carcinomas behave similarly to their aggressive pulmonary counterpart, small cell lung carcinoma. Most patients with gastroenteropancreatic neuroendocrine tumors present with symptoms of metastasis, most commonly to the liver. There have been no case reports, to our knowledge, until now that demonstrate metastasis to the central nervous system.

**Case presentation:**

A 72-year-old male with poorly-differentiated stage IIIB neuroendocrine carcinoma of the colon presented with acute altered mental status and right facial droop. Head CT was negative for an acute hemorrhagic process without evidence of suspicious lesions. Several days later, the patient developed fever and neck stiffness suspicious for bacterial meningitis. A lumbar puncture procedure was performed. Cytology of the CSF demonstrated metastatic disease to the central nervous system and the final diagnosis of carcinomatous meningitis secondary to metastatic neuroendocrine carcinoma of the colon was made.

**Conclusions:**

High-grade gastroenteropancreatic neuroendocrine carcinomas most commonly metastasize to the liver, which often corresponds with the patient’s initial presentation. When neuroendocrine tumors do metastasize to the central nervous system, the primaries are most commonly of pulmonary origin. When meningeal metastasis does occur, it commonly presents as neurologic deficits or cerebrovascular events, rarely does meningeal metastasis mimic bacterial meningitis with symptoms of fever, photophobia and meningismus. As neuroendocrine carcinomas have been increasing in incidence over the past several decades, it is important to consider varying metastatic presentations when working up a patient with a diagnosis of neuroendocrine tumor.

## Background

Neuroendocrine tumors represent an expansive group of neoplasms that share an etiology of epithelial origin with neuroendocrine differentiation and potential for hormone release. In the past two decades, the World Health Organization has re-classified neuroendocrine tumors with emphasis on their morphology and proliferation rates. The neuroendocrine tumors of the gastrointestinal tract, pancreas and liver are now recognized as either well-differentiated neuroendocrine tumors (NETs) or poorly-differentiated neuroendocrine carcinomas (NECs) [1]. Poorly-differentiated NECs behave similarly to their aggressive pulmonary counterpart, small cell lung carcinoma. While symptoms of gastroenteropancreatic (GEP) NECs often relate to the primary origin, one analysis of 2546 poorly-differentiated NECs demonstrated that up to 57% of patients presented with symptoms related to metastasis rather than the primary site [2]. Metastases most commonly are to the liver, followed by bone, lung and rarely the central nervous system [3].

## Case report

A 72-year-old right handed male diagnosed with poorly-differentiated, stage IIIB neuroendocrine carcinoma of the colon s/p hemicolectomy, small bowel resection and carboplatin-etoposide × 3 cycles presented to the emergency department with acute altered mental status and right facial droop.

Four months prior, he presented with constipation and anemia. Colonoscopy revealed a large raised flat lesion in the transverse colon and CT abdomen demonstrated RLQ mesenteric lymphadenopathy. He underwent right hemicolectomy and small bowel resection weeks later. Pathology was significant for poorly-differentiated grade 3, neuroendocrine carcinoma with focal lymphovascular invasion and tumor invasion through the muscularis propria into the subserosa. Margins were negative, no perineural invasion and 1/33 lymph nodes were positive for carcinoma. There was an absence of non-neuroendocrine component. Immunohistochemical stains were positive for: AE1/AE3, CD56, chromogranin, and synaptophysin; Ki-67 of 60% proliferative index. He was staged as pathologic T3N1a, stage IIIB.

**Fig. 1:**
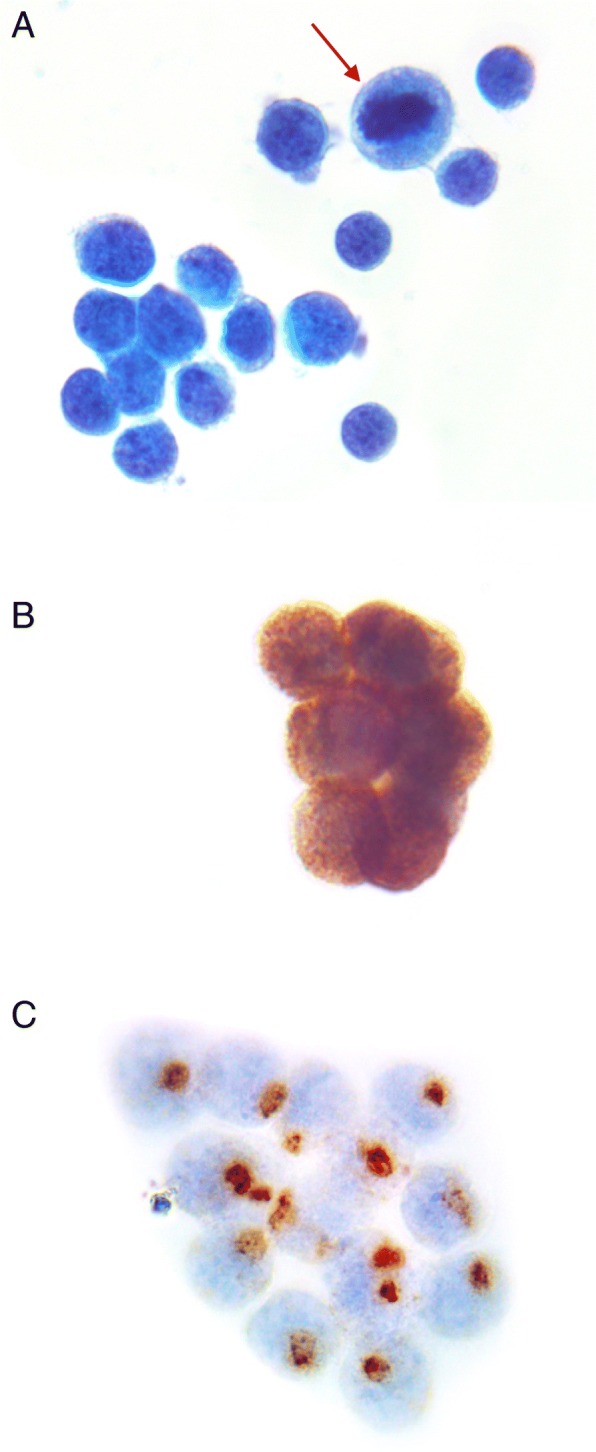
Lumbar puncture cytology. **a** Papanicolaou stain of CSF showing an atypical population of tumor cells with stippled chromatin and mitotic figures readily identified (arrow). **b** Cluster of tumor cells showing positive granular cytoplasmic staining with synaptophysin and (**c**) Cluster of tumor cells showing positive perinuclear staining with Cytokeratin AE1/AE3 in a typical “dot pattern”

In the emergency department, head CT was negative for an acute hemorrhagic process and did not demonstrate any suspicious lesions. Within one day of admission, the facial droop resolved. Further imaging, CT chest abdomen pelvis, revealed stable enlarged mediastinal lymphadenopathy and a subcentimeter retroperitoneal lymph node but no progression was evident. Two days into the hospital stay, the patient developed fever and subsequently neck stiffness. His chest x-ray and urinalysis were non-diagnostic; EEG showed diffuse slowing but no seizure activity. A lumbar puncture was performed with cytopathology of the CSF suggesting metastatic disease to the central nervous system (Fig. 1), along with lymphocytic pleocytosis, normal glucose, and significantly elevated protein and lactic acid. Cytologic analysis showed tumor cells with characteristically-high nuclear to cytoplasmic ratio, relatively round nuclei with stippled “salt and pepper” nuclear chromatin and minimal cytoplasm, features consistent with metastatic neuroendocrine carcinoma (Fig. 1a). Immunohistochemistry showed the tumor cells were strongly positive for synaptophysin (Fig. 1b) and Cytokeratin AE1/AE3 (Fig. 1c) with a typical perinuclear dot pattern.

Medical oncology and radiation oncology were consulted. No further chemotherapy was recommended as he had progressed after completing 3 of 6 cycles of carboplatin-etoposide. Enrollment in an erlotinib trial was discussed vs palliative therapy. While radiation was considered to be potentially palliative for his symptoms, radiation oncology deemed it would be unlikely to change his overall survival. He was started on palliative high-dose steroids and ultimately transferred to inpatient hospice care. The patient passed away within a week of entering hospice.

## Discussion

This case depicts the rapid progression of a high-grade neuroendocrine carcinoma of the colon with metastases to the meninges causing atypical symptoms of carcinomatous meningitis.

The most common site for metastasis of NECs of the GI tract is the liver, with up to 57% of tumors presenting with metastasis upon initial presentation [2]. Other sites of metastasis for GEP NEC at presentation include peritoneum (15.2%), soft tissue (12.3%), lung (10.5%) and bone (10.3%) [4]. Between 1.5 and 10.3% of patients with NET have brain metastasis, but the majority of these cases involve NETs of pulmonary origin [5, 6]. The presentation of brain metastasis has been studied in other neuroendocrine tumors including carcinoid tumor. In one study of 1633 patients with carcinoid tumors, 1.5% (*n* = 24) developed brain metastasis, while the most common sites of metastasis were lymph nodes (52%) and liver (39%) [7]. A retrospective study completed in 2016 analyzed the presentation of neuroendocrine tumors with metastases to the brain [6]. Regarding the location of the primary lesions, the overwhelming majority (84%, *n* = 24/31) were of pulmonary origin with other primaries located in the breast, esophagus, lymph nodes and uterus. Though case reports of metastatic brain neuroendocrine tumors originating from the pancreas and cervix have been reported, there are none to our knowledge of primaries originating in the large intestine [3, 8].

In addition to the uncommon primary and metastatic location of this neuroendocrine carcinoma, the case is also intriguing due to its atypical clinical presentation. When meningeal metastasis occurs, most commonly from breast cancer, lung cancer, or melanoma, it often presents with symptoms of neurologic dysfunction related to the occupied area of metastases [9]. Rhun and colleagues describe the typical metastatic central nervous system presentation stemming from invasion or compression of neuronal tracts resulting in neurologic deficits and focal symptoms, or symptoms from invasion and compression of blood vessels resulting in cerebrovascular events. Resulting symptoms can be divided into cerebral, cranial nerve, and spinal cord/root dysfunction. Cerebral involvement can present as headache, acute change in mental status, ataxia, nausea, or vomiting. Cranial nerve involvement frequently causes diplopia or facial paresis. Lastly, spinal complications include lower motor neuron weakness, limb paresthesia, back or neck pain, and radiculopathy [9].

While the patient did experience a facial droop that may be attributable to facial nerve involvement or a posterior circulation transient ischemic attack, the progression of his illness to include fever and nuchal rigidity is extremely unusual for the presentation of carcinomatous meningitis. Few reports have been documented in patients with meningeal carcinomatosis that demonstrate symptoms such as meningismus, fever, and photophobia mimicking acute bacterial meningitis. Due to this historically uncommon presentation, literature is limited to case reports surrounding acute meningitis in relation to recurrent NETs.

Moreover, a unique presentation of carcinomatous meningitis from NEC is exhibited in this patient with the uncommon primary location in the colon. To our knowledge, this is the first report of meningeal metastasis from a neuroendocrine tumor of the large intestine. While still a relatively rare tumor, incidence of gastrointestinal neuroendocrine tumors has significantly risen since the 1970s; it is therefore important to be aware of the common presenting symptoms of neuroendocrine tumors [10]. Though liver metastasis is the most common site for initial metastasis of GEP NEC, it is important to keep in mind the potential for metastasis to the CNS when evaluating a patient with NEC; neurological workup may be warranted. Additionally, while cranial nerve palsies and cerebrovascular events are more typical indications of meningeal metastases, patients may present with less-common symptoms, including those that are more often associated with acute bacterial meningitis.

## Abbreviations

CNS: Central nervous system
GEP: Gastroenteropancreatic
NEC: Neuroendocrine carcinoma
NET: Neuroendocrine tumor
